# Treatment of hepatocellular carcinoma with hepatic vein tumor thrombosis protruding into the inferior vena cava by conversion surgery following chemotherapy with regorafenib: a case report

**DOI:** 10.1007/s12328-019-01077-4

**Published:** 2020-01-22

**Authors:** Kazuhisa Takeda, Yuji Tsurumaru, Yuji Yamamoto, Kentaro Araki, Yu Kogure, Koichi Mori, Kazuya Nakagawa, Tetsuya Shimizu, Goro Matsuda, Hitoshi Niino, Hitoshi Sekido, Satoshi Kobayashi, Manabu Morimoto, Chikara Kunisaki, Itaru Endo

**Affiliations:** 1 Department of Gastroenterological Center, Yokohama City University Medical Center, 4-57 Urafune-cho, Minami-ku, Yokohama, Kanagawa 232-0024 Japan; 2 Department of Surgery, Yokohama Medical Center, 3-60-2 Harajyuku, Totsuka-ku, Yokohama, 245-8575 Japan; 3 Department of Pathology, Yokohama Medical Center, 3-60-2 Harajyuku, Totsuka-ku, Yokohama, 245-8575 Japan; 4 Division of Hepatobiliary and Pancreatic Medical Oncology, Kanagawa Cancer Center, 2-3-2 Nakao Asahi-ku, Yokohama, Kanagawa 241-8515 Japan; 5 Department of Gastroenterological Surgery, Yokohama City University Graduate School of Medicine, 3-9 Fukuura, Kanazawa-ku, Yokohama, 236-0004 Japan

**Keywords:** Hepatocellular carcinoma, IVC-HVTT, Regorafenib

## Abstract

Regorafenib is an oral multikinase inhibitor affecting angiogenesis, oncogenesis, metastasis, and tumor immunity. As a systemic treatment, it has been shown to provide survival benefits in hepatocellular carcinoma (HCC) patients progressing on sorafenib treatment. We report herein a case of HCC with hepatic vein tumor thrombosis protruding into the inferior vena cava (IVC-HVTT) which was successfully treated by surgery following second-line chemotherapy with regorafenib. A 79-year-old man with chronic hepatitis was diagnosed with HCC. Computed tomography revealed a solitary tumor in segments 7 and 8 and an IVC-HVTT from the right hepatic vein. Since IVC-HVTT removal is a difficult procedure, the tumor was diagnosed as unresectable, and administration of sorafenib was started. Five weeks later, the lesion had increased in size by 15.3%; subsequently, regorafenib was given as second-line therapy for 12 months. After shrinkage of the IVC-HVTT, the patient was referred to our hospital for surgery. One month after the cessation of regorafenib, an extended resection of segment 8 and total removal of the IVC-HVTT was successfully performed without using total hepatic vascular exclusion. There were no serious postoperative complications. Additionally, there has been no recurrence for about 2 years since the initial therapy.

## Introduction

The treatment strategy for hepatocellular carcinoma (HCC) is well established by the American Association for the Study of Liver Diseases [1] as well as the European Association for the Study of the Liver-European Organization for Research and Treatment of Cancer (EASL-EORTC) guidelines [2]. While surgical resection, ablation, or transplantation are potential curative options for early-stage HCC, transcatheter arterial chemoembolization is recommended for intermediate-stage HCC. Sorafenib is recommended for advanced-stage HCC which involves vascular invasion or extrahepatic spread [3]. Sorafenib is recognized as the first line of chemotherapy for systemic treatment [3, 4]. However, in cases where the efficacy of sorafenib is not confirmed, the use of regorafenib as second-line chemotherapy is recommended [4]. Regorafenib has been approved for unresectable/advanced colorectal cancer and gastrointestinal stromal tumors [5, 6]. In addition, its efficacy has been demonstrated for patients who progressed on sorafenib treatment. In Japan, insurance covers the treatment of unresectable HCC exacerbated after cancer chemotherapy. However, there have been no reports of conversion surgery for HCC performed after the use of regorafenib as second-line chemotherapy. We report a case of locally advanced unresectable HCC with hepatic vein tumor thrombosis protruding to the inferior vena cava (IVC-HVTT), which was treated successfully by conversion surgery following second-line chemotherapy with regorafenib.

## Case report

**Fig. 1:**
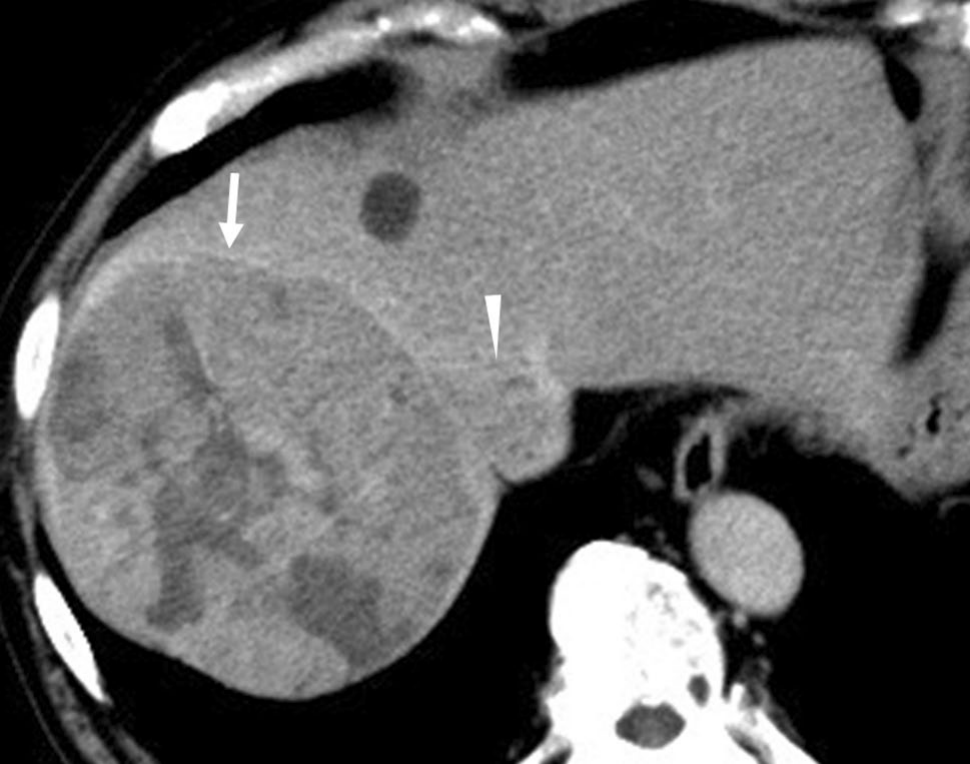
CT findings at initial diagnosis. Shown are the CT images from the patient’s first visit to his previous hospital. A solitary tumor, 13 × 9.5 cm in diameter, can be seen in segments 8 and 7 (arrow). IVC-HVTT, 18 × 20 mm in diameter (arrowhead), can be clearly seen from the right hepatic vein. *CT* computed tomography, *IVC-HVTT* hepatic vein tumor thrombosis protruding into the inferior vena cava

**Fig. 2:**
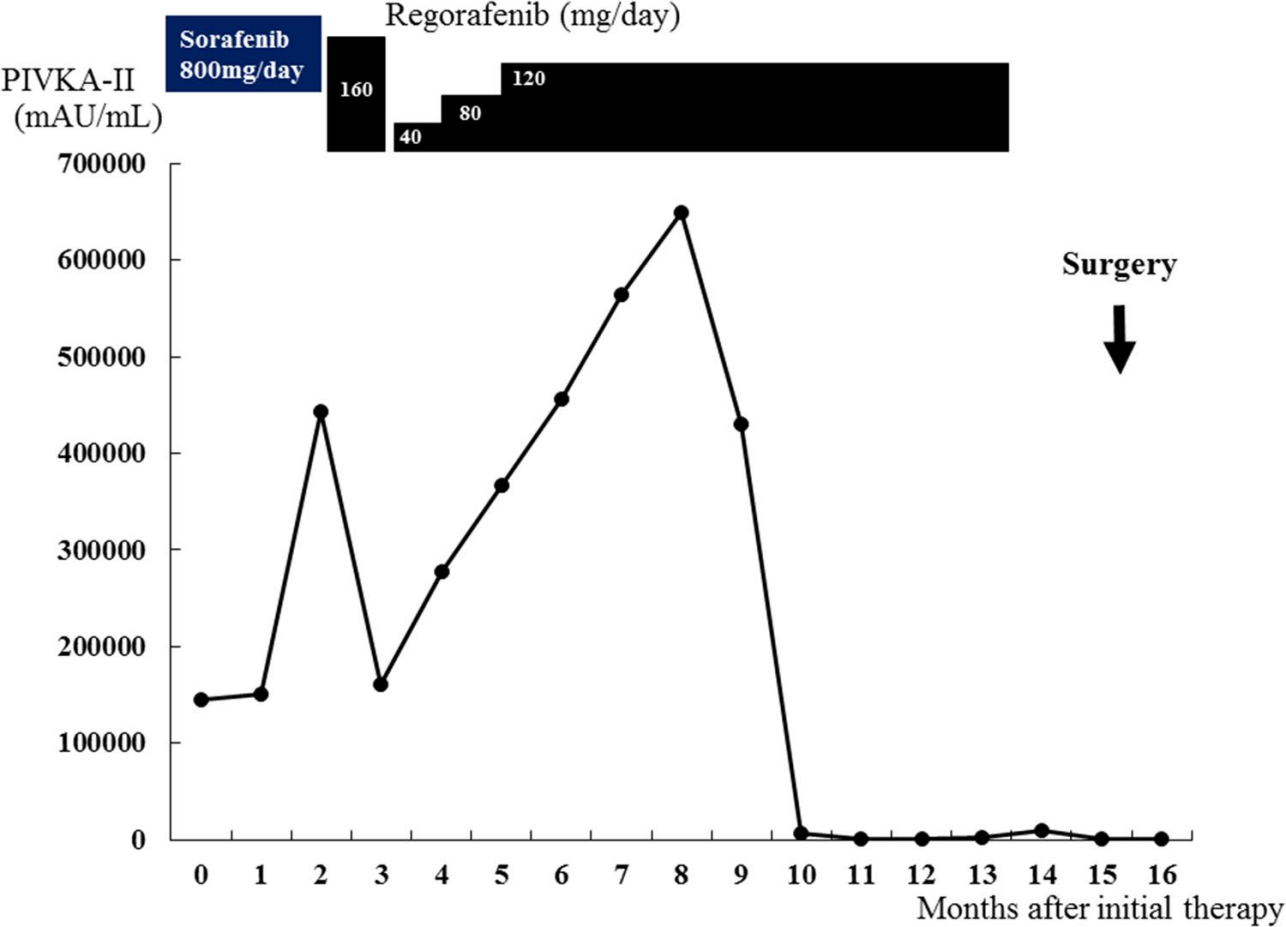
Timeline of the therapeutic modalities and changes in levels of protein induced by vitamin K absence/agonist-II (PIVKA-II)

**Fig. 3:**
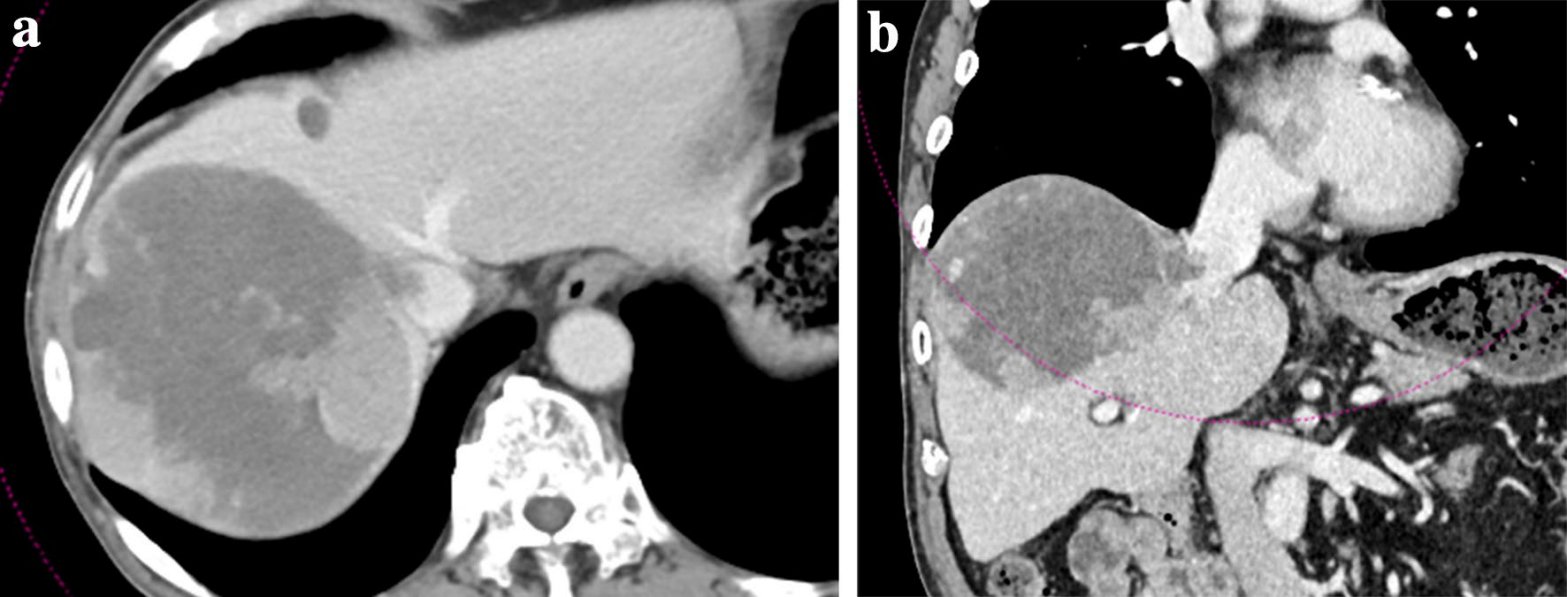
CT findings after 10 months of regorafenib treatment. Shown are the tumor characteristics from the **a** horizontal and **b** frontal plane. Though graded as stable disease, an 18.6% reduction in tumor size and shrinkage of the IVC-HVTT can be seen. *CT* computed tomography, *IVC-HVTT* hepatic vein tumor thrombosis protruding into the inferior vena cava

A 79-year-old man with hepatitis B virus-associated chronic hepatitis was diagnosed with HCC at his previous clinic. Computed tomography (CT) revealed a solitary tumor, 13 × 9.5 cm in diameter, located in segments 7 and 8. In addition, an IVC-HVTT, 18 × 20 mm in diameter, was detected from the right hepatic vein (Fig. 1). The tip of the tumor thrombosis did not extend beyond the diaphragm but occupied the entire IVC lumen. Distant metastasis was not observed. Based on the 8th Union for International Cancer Control classification of HCC, the tumor was graded as T4N0M0 and stage III B. The following tumor markers were detected: alpha fetoprotein (3.8 ng/mL) and protein induced by vitamin K absence/agonist-II (PIVKA-II) (145,000 mAU/mL). The patient was diagnosed with unresectable HCC at his previous hospital because total hepatic vascular exclusion (THVE) would be necessary for the removal of the IVC-HVTT and it was judged technically difficult to perform; in addition, the clinical benefit of resection therapy was doubtful. Therefore, administration of sorafenib (800 mg/day) was started as first-line chemotherapy. Lenvatinib was not available at that time because it was not covered by insurance. Five weeks of sorafenib administration (total 23.2 g) resulted in stable disease (SD) based on the Response Evaluation Criteria in Solid Tumors (RECIST). However, the tumor size had increased by 15.3%, and the PIVKA-II levels had increased to 444,000 mAU/mL (Fig. 2). Based on these outcomes, sorafenib was judged to be ineffective, and regorafenib was started as second-line chemotherapy. Regorafenib was started at 160 mg; however, grade 3 liver dysfunction appeared based on the Common Terminology Criteria for Adverse Events (version 4.0), and this drug was withdrawn for 1 week. Thereafter, it was restarted at 40 mg, increased to 120 mg, and maintained. It was administered for 12 months (total 28.74 g) and resulted in stage III B SD. The tumor and IVC-HVTT decreased by 18.6% and 56%, respectively (Fig. 3). PIVKA-II levels also decreased from the maximum 649,000 mAU/mL to 2420 mAU/mL (Fig. 2). Furthermore, CT revealed that 75.4% of the tumor became unstructured at the core, and the outcome was evaluated as partial response (PR) based on the modified RECIST (mRECIST) [7]. However, grade 2 anorexia occurred during the course of the treatment, and the patient did not wish to continue with the chemotherapy. Therefore, he was referred to our hospital for conversion surgical treatment.

**Fig. 4:**
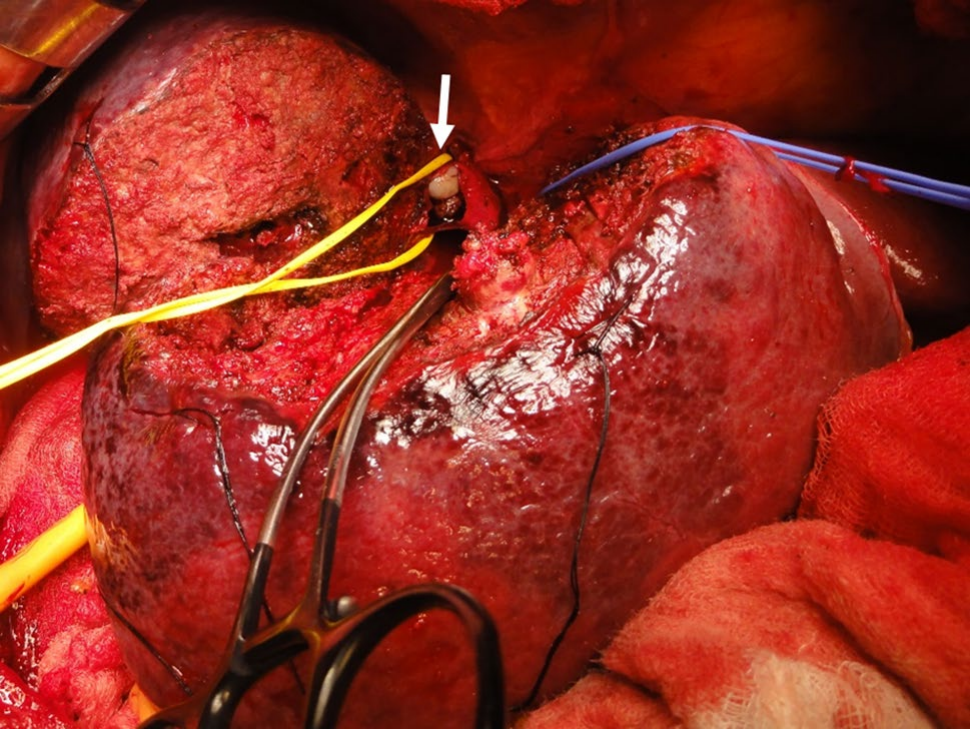
Resection of the IVC-HVTT. Shown is the extended resection of segment 8, including partial resection of segments 7 and 1, and total removal of the IVC-HVTT. For the removal of the IVC-HVTT, the IVC was clamped in half at a root of the right hepatic vein (arrow). *IVC-HVTT* hepatic vein tumor thrombosis protruding into the inferior vena cava

**Fig. 5:**
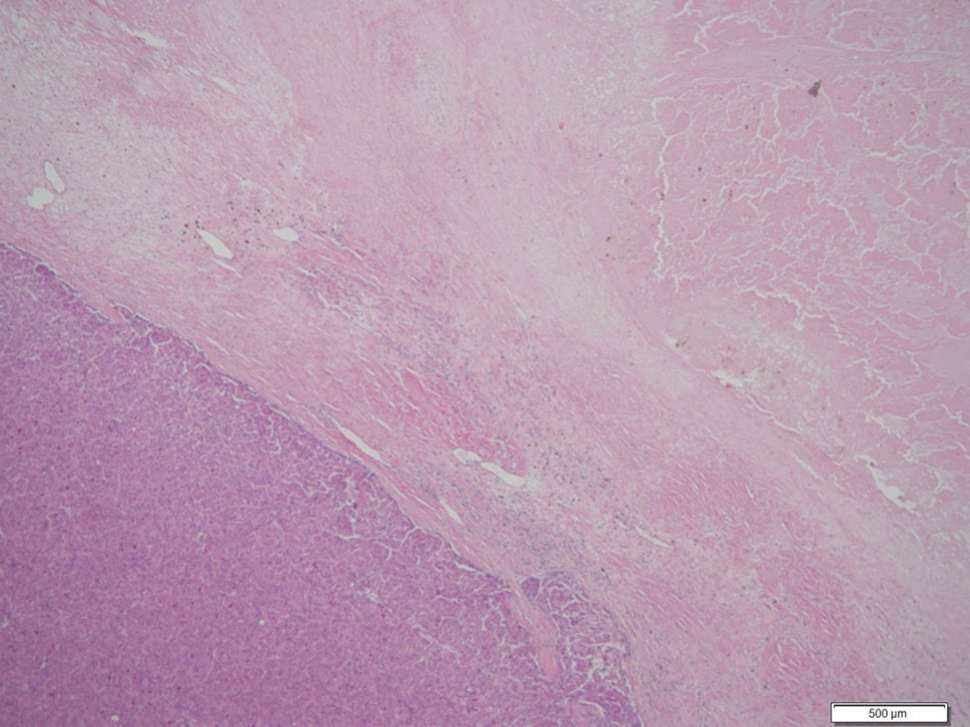
Histological findings from the main tumor (hematoxylin and eosin stain). The main tumor of the liver shows only 20% of viable cancer cells

At his first visit to our hospital, the patient’s Eastern Cooperative Oncology Group performance status was 0. The preoperative liver-function tests showed the following: total bilirubin, 0.5 mg/dL; albumin, 3.4 g/dL; prothrombin test, 1.06 INR; and indocyanine green retention rate at 15 min (ICGR15): 32.63%. The Child–Pugh score was A with 6 points, and the liver damage score was B. Blood tests revealed (1) peripheral white blood-cell count: 5,900/mm^3^, (2) neutrophils: 3670/mm^3^, (3) platelets: 21.6 × 10^3^/mm^3^, and (4) C-reactive protein: 1.93 mg/dL. One month after the cessation of regorafenib, an extended resection of segment 8 including partial resection of segments 7 and 1 and total removal of the IVC-HVTT were performed. An intraoperative transesophageal echo was used for monitoring the pulmonary embolism caused by the IVC-HVTT. For the removal of the IVC-HVTT, the IVC was clamped in half, and the use of THVE was avoided (Fig. 4). The duration of the surgery was 318 min and involved 650 mL of intraoperative hemorrhage without blood transfusion. There were no serious postoperative complications, and the patient was discharged on day 16 after the surgery. The PIVKA-II level dropped and was within the normal range after the operation. The resected specimen had 20% viable cancer cells in the main tumor of the liver and 30% in the tumor thrombus (Fig. 5). The resected margin of the cut surface of the liver did not show any cancer cells, indicative of potentially curative resection. It has been 2 years since the initial therapy, and the patient is surviving with no recurrence for 8 months following the hepatectomy.

## Discussion

The incidence of HCC with IVC-HVTT is only about 1.4% based on Japanese nationwide surveillance [8]. Generally, HCC associated with macroscopic vascular invasion is regarded as an advanced stage of the disease [9]. For patients with HCC accompanied by vascular invasion, embolization, hepatectomy, hepatic arterial infusion chemotherapy, and molecular targeted therapy are recommended. Each treatment is selected according to the individual situation, i.e., liver function, the condition of HCC, and the extent of vascular invasion. Because it is currently difficult to provide a universal ranking for these four treatments, they are recommended in parallel with the treatment for HCC accompanied by vascular invasion [10]. For our case, a molecular targeted drug was selected. Sorafenib as well as lenvatinib are recommended as the first-line therapy for unresectable advanced HCCs; however, only sorafenib could be used at that time. In contrast, following the recent advances in surgical techniques and perioperative management, surgical resection for IVC-HVTT has been proposed [8]. However, the median survival rate after resection is reported to be 1.37 years, which is comparable to that following chemotherapy [8]. Therefore, instead of straightforward hepatectomy, effective perioperative therapy is recommended in such cases. Furthermore, the 90-day mortality rate following resection of HCC with IVC-HVTT is as high as 9.9% [8]. This high mortality rate may be related to the use of THVE. Although THVE is needed for the resection of liver tumors involving IVC [11], it is technically complicated and may cause liver damage due to the prolonged ischemia and circulatory instability caused by the absence of venous return via the IVC [11]. These conditions cause congestion of the kidneys and intestine, which may explain why the damage and morbidity after THVE are much greater than under inflow occlusion alone [12]. Therefore, it is better to remove the tumor completely without THVE. For these reasons, HCC in our patient was diagnosed as unresectable at first and was treated with sorafenib followed by regorafenib. Although regorafenib and ramucirumab are recommended as second-line therapy for HCC [13], the only regorafenib was available at that time. Following the regorafenib treatment, the IVC-HVTT decreased in size, eliminating the need for THVE during hepatectomy.

Regorafenib is an oral multikinase inhibitor that blocks the activity of protein kinases involved in angiogenesis, oncogenesis, metastasis, and tumor immunity [4]. It also induces apoptosis in hepatocytes [14]. Though the pharmacological activity of regorafenib is similar to that of sorafenib, the former has been shown to be effective in HCC that does not respond to sorafenib treatment [4]. One of the underlying mechanisms that account for this difference in efficacy is the inhibition of tyrosine kinase with immunoglobulin-like and epidermal growth factor-like homology domain 2 (TIE2) activity by regorafenib and not sorafenib [15]. TIE2, a receptor expressed on vascular endothelial cells, is activated by binding to angiopoietin 2 and contributes to angiogenesis. Furthermore, monocytes expressing TIE2 have been reported to have tumor angiogenic activity [16]. The inhibition of TIE2 reduces TIE2-expressing monocytes in HCC and angiogenesis [17]. Therefore, the sequential use of sorafenib and regorafenib is effective in HCC where the influence of tumor vessel application by TIE2 is large.

The median survival in HCC patients treated with regorafenib is only 10.6 months [4]. Conversion surgery is the next-best strategy to improve the long-term survival in these patients. Recent studies have shown that the 5-year survival rates after downstaging followed by conversion hepatectomy are comparable to those after primary liver resection [18]. One study has reported survival for 4 years after a multidisciplinary therapy, including first-line sorafenib and subsequent conversion surgery [19]. However, there are no reports on conversion surgery after second-line chemotherapy using regorafenib for HCC that was initially diagnosed as unresectable. According to previous reports of hepatectomy after sorafenib, the drug was interrupted for 7–45 days from hepatectomy and no adverse effects of preoperative administration of sorafenib were observed during and immediately after hepatectomy for HCC [19, 20]. Based on these reports, regorafenib was suspended for 1 month before hepatectomy in our case.

Although the tumor could not be downstaged based on the RECIST evaluation, tumor size and PIVK-II level decreased following regorafenib treatment in our case. Furthermore, after chemotherapy, 75.4% of the tumor became unstructured and was evaluated as PR based on mRECIST evaluation. In fact, 70–80% of the tumor appeared necrotic microscopically. These CT-based morphological changes were reported to have a statistically significant association with pathologic response and overall survival [21]. Although the prognosis of HCC patients with IVC-HVTT is poor, in patients who respond well to chemotherapy, conversion surgery could help to improve their long-term survival. A large-scale observational study to evaluate the benefits of conversion surgery should, therefore, be considered for such patients who respond well to chemotherapy.

In conclusion, we reported a case of locally advanced HCC which was treated with conversion surgery after regorafenib as second-line chemotherapy. The shrinkage of IVC-HVTT by regorafenib treatment was essential for conversion surgery; however, our case may have been uniquely suited to this treatment because the intrahepatic HCC was solitary and IVC-HVTT extension into the vein was relatively limited. However*,* multidisciplinary therapy, including conversion surgery, may be an effective strategy for treating HCC diagnosed as unresectable and for improving long-term survival.
